# Hsa_circ_0004662 Accelerates the Progression of Ulcerative Colitis via the microRNA‐532/HMGB3 Signalling Axis

**DOI:** 10.1111/jcmm.70430

**Published:** 2025-03-18

**Authors:** Chunhua Qiu, Yun Chen, Huan Xia, Jun Duan, Lu Zhang, You Zhang, Ziyang Chen, Li Zhang

**Affiliations:** ^1^ Department of Gastroenterology, Sichuan Provincial People's Hospital, School of Medicine University of Electronic Science and Technology of China Chengdu China; ^2^ Department of Geriatric Gastroenterology, Sichuan Provincial People's Hospital, School of Medicine University of Electronic Science and Technology of China Chengdu China; ^3^ Geriatrics Research Institute, Sichuan Provincial People's Hospital, School of Medicine University of Electronic Science and Technology of China Chengdu China

**Keywords:** Circ_0004662, HMGB3, inflammation, miR‐532, ulcerative colitis

## Abstract

Increasing research has indicated that circular RNAs (circRNAs) are crucial for the development of ulcerative colitis (UC). Thus, we attempted to identify the role of hsa_circ_0004662 in UC progression. Hsa_circ_0004662 expression was determined via qRT‐PCR. Lipopolysaccharide (LPS)‐induced inflammation in normal colonic epithelial cells (ECs). The hsa_circ_0004662 content was then assessed in a mucosal inflammatory bowel disease (IBD) model. Cell proliferation was examined via CCK‐8 and EdU uptake assays. Apoptotic rates were analysed via flow cytometry. The protein content was quantified via Western blotting. Enzyme‐linked immunosorbent assay kits were used to detect IL‐1β, TNF‐α and IL‐6, and dual‐luciferase reporter (DLR) assays were used to identify interactions between miR‐532 and circ_0004662 or HMGB3. An animal model of UC was also developed for confirmation. In this study, we identified the function of hsa_circ_0004662 in promoting UC progression. Hsa_circ_0004662 was upregulated in clinical UC tissues and LPS‐induced colonic ECs, and its knockdown inhibited apoptosis, reduced inflammatory cytokine release and promoted cell proliferation in vitro. Mechanistically, hsa_circ_0004662 acted as a molecular sponge for miR‐532, which targets HMGB3. The hsa_circ_0004662/miR‐532/HMGB3 axis was further validated in a DSS‐induced colitis mouse model, where hsa_circ_0004662 knockdown attenuated inflammation and tissue damage. These findings suggested that hsa_circ_0004662 contributes to UC progression through the miR‐532/HMGB3 signalling pathway, offering potential targets for UC therapy.

## 1 Introduction

Ulcerative colitis (UC) is a severe inflammatory bowel disease (IBD) characterised by relapsing and retreatment episodes. Its prevalence is increasing in developing countries owing to the widespread adoption of Western diets globally [1, 2]. This condition is characterised by ulcers and inflammation in the colon and rectum regions, leading to gastrointestinal complications such as diarrhoea, rectal bleeding and stomach pain [3, 4]. UC progression is intricate and comprises various environmental, genetic and immunological variables [2]. Disruption of intestinal epithelial cells (IECs) and abnormalities in the epithelial barrier are commonly recognised as critical factors in severe inflammation [5]. High IEC mortality impairs host–microorganism homeostasis, mucosal immune control, nutrient circulation and intestinal barrier integrity, leading to recurrent chronic colitis [6]. Investigating the root cause and underlying mechanism of IEC mortality may lead to the development of appropriate treatment options for UC patients.

Circular RNAs (circRNAs) are a newly emerged type of covalently closed noncoding RNA generated via reverse splicing of the 3′ and 5′ ends [7, 8]. Owing to their structural properties, they are expressed primarily, with remarkable stability and a high degree of conservation. Indeed, circRNAs, which originate from exonic, intergenic or intronic parts of the genome, frequently present expression patterns specific to tissue type and developmental stage [9]. They can act as scaffolds for protein complexes, thereby regulating gene expression, such as that of their parental genes. Furthermore, they modulate alternative splicing events, regulate binding amongst proteins and RNAs and act as molecular sponges by sequestering miRNAs in a specific sequence [10]. Recent studies on miRNAs in UC have highlighted the significant involvement of miRNAs, including miR‐182‐5p [11] and miR‐29c‐3p, [12] in UC progression. Hsa_circ_0004662 is a newly discovered circRNA that reportedly enhances osteoarthritis (OA) occurrence via miR‐424‐5p/VEGFA pathway modulation [13]. Despite the reported significance of hsa_circ_0004662 in OA, the process by which it is regulated in UC remains unclear.

The HMGB3 protein belongs to the HMGB protein class, a subfamily of nuclear proteins that exhibit increased electrophoretic mobility. Multiple reports have suggested that the HMGB3 gene is crucial in the development of malignant diseases. Recently, several microRNAs and long noncoding RNAs have been shown to regulate HMGB3 expression. These RNAs either inhibit target RNA translation or activate RNA degradation. Conversely, the function of HMGB3 and its associated mechanisms in UC has not yet been elucidated.

Therefore, more research into the underlying mechanisms of UC is necessary to advance new therapeutic approaches with significant practical implications. Overall, we attempted to identify the significance of hsa_circ_0004662 in UC modulation via the miR‐532/HMGB3 axis and identified undetected biomarkers relevant to UC treatment.

## 2 Materials and Methods

### 2.1 Patients and Sample Acquisition

All samples (human colorectal mucosal tissues) were derived from healthy volunteers (*n* = 29) and UC patients (*n* = 47). All participants underwent endoscopic examination at the Department of Geriatric Gastroenterology, Sichuan Provincial People's Hospital, School of Medicine, University of Electronic Science and Technology of China. Ethical approval was obtained from the appropriate institutional board, and the study adhered to the guidelines of the Declaration of Helsinki (2013 revision). Informed consent was obtained from individual participants prior to tissue sample collection. These samples were preserved in liquid nitrogen for further analysis.

### 2.2 Animals and Experimental Design

The same method was used to establish an animal model as previously described [14]. The animals included male Kunming (KM) mice (20 ± 2 g) obtained from the respective institute. The Animal Care and Use Committee of the participating institution provided approval for all the animal protocols [Lunshen(Yan)2022‐388]. All the mice were housed in a standard environment with 20% humidity, 22°C–24°C and a 12 h light/dark cycle. The animals had free access to a standard diet and water. Following 1 week of acclimation, 20 mice were arbitrarily assigned to 4 cohorts (*n* = 5). Cohort 1 received only drinking water, whereas the other cohorts (Cohorts 2–4) were provided with a 3% solution of DSS (Mw 36,000–50,000 kDa, MP Biomedicals LLC, USA) mixed in their drinking water over a 7‐day period for UC induction. In Cohort 3, control lentivirus (LV‐sh‐CTRL, GenePharma, Suzhou, China) (100 μL) was injected into the tail vein twice/week, whereas in Cohort 4, circ_0004662‐knockdown lentivirus (LV‐sh‐circ_0004662, GenePharma) (100 μL) was used via *the* same route and time. All the mice were euthanized after 14 days via *the* intraperitoneal injection of 1% sodium pentobarbital. Colon tissues were harvested, and their lengths were estimated. They were immediately washed with physiological saline (chilled). These samples were promptly fixed in 10% formalin, whereas the other samples were preserved at −80°C for further examination.

### 2.3 Statistical Analysis

Statistical analyses were performed in SPSS 18.0 (SPSS Inc.). For intercohort comparisons, t‐tests and one‐way ANOVAs were used, followed by Dunnett's post hoc tests. The associations between circ_0004662, miR‐532 and HMGB3 expression were examined via Pearson's correlation coefficient. *p* < 0.05 represented the significance threshold. The data were analysed in triplicate, and the results are presented as the means ± SDs.

## 3 Results

### 3.1 Hsa_circ_0004662 is Upregulated in UC

**FIGURE 1:**
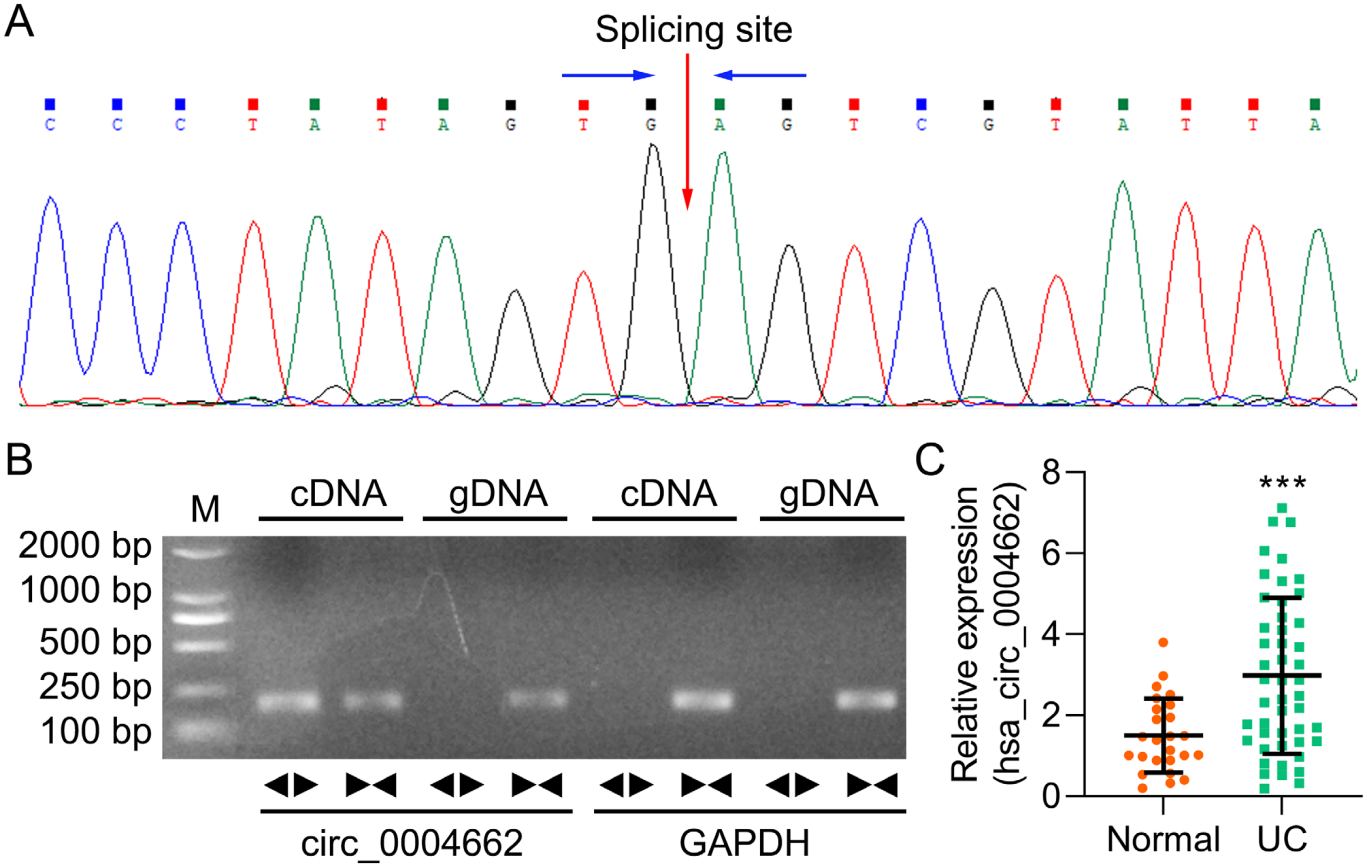
Hsa_circ_0004662 is upregulated in UC tissues. Examination of the sequencing data (A) and the PCR product of cric_0004662 via agarose gel (1.5%) electrophoresis (B). (C) Level of circ_0004662 in paired UC and normal samples (*n* = 47). All the data are presented as independent triplicate experiments and are presented as the means ± SDs; ***p < 0.001.

First, the circRNA signature of circ_0004662 was identified, as illustrated in Figure 1A,B, and the circular format of hsa_circ_0004662 was identified via convergent and divergent primers. Furthermore, the circ_0004662 contents in the UC and corresponding normal tissues were examined via qRT‐PCR. We revealed that the circ_0004662 content was typically elevated in UC biopsies compared with healthy tissue (Figure 1C). These results confirmed that circ_0004662 was upregulated in UC tissue.

### 3.2 Hsa_circ_0004662 Downregulation Inhibits LPS‐Induced Apoptosis and Inflammatory Cytokine Release in Colon ECs

**FIGURE 2:**
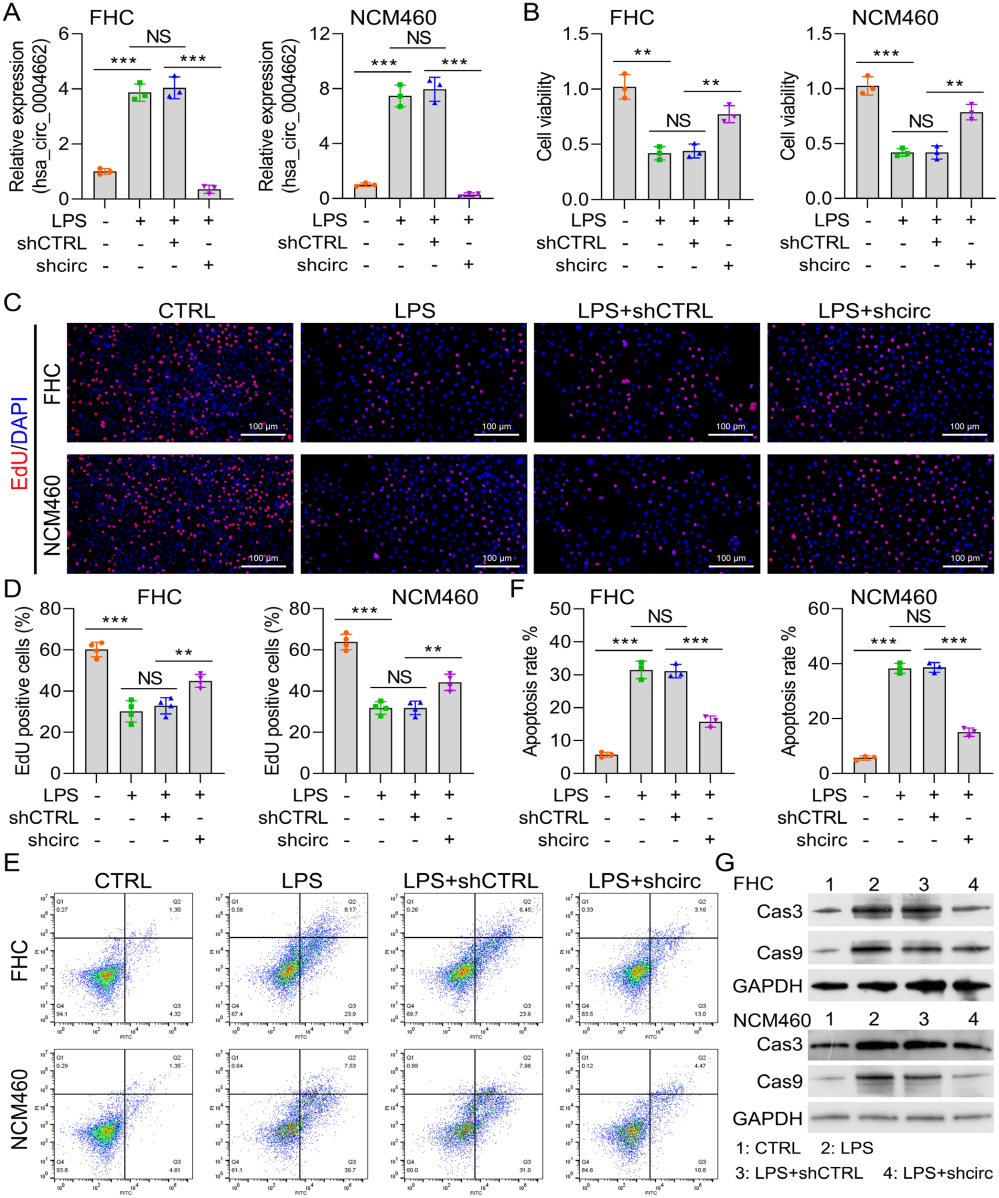
Hsa_circ_0004662 downregulation inhibits LPS‐induced apoptosis. (A) RT‐qPCR assessment of the hsa_circ_00046622 content in LPS‐, LPS + shCTRL‐ and LPS + shcirc‐treated FHC and NCM460 cells. (B) Cell viability was evaluated via the CCK‐8 test (C, D). Cell proliferation detection via *the* EdU assay. Scale bar (100 μm). Relative to the control group. (E, F) After quantitative analysis, FC was used to assess FHC and NCM460 cell apoptosis. (G) WB analysis of the expression of Caspase 3 and 9 in FHC and NCM460 cells exposed to LPS, LPS + shCTRL or LPS + shcirc. The data are presented as the means ± SDs (*n* = 5); ****p < 0.001, **p < 0.01.

**FIGURE 3:**
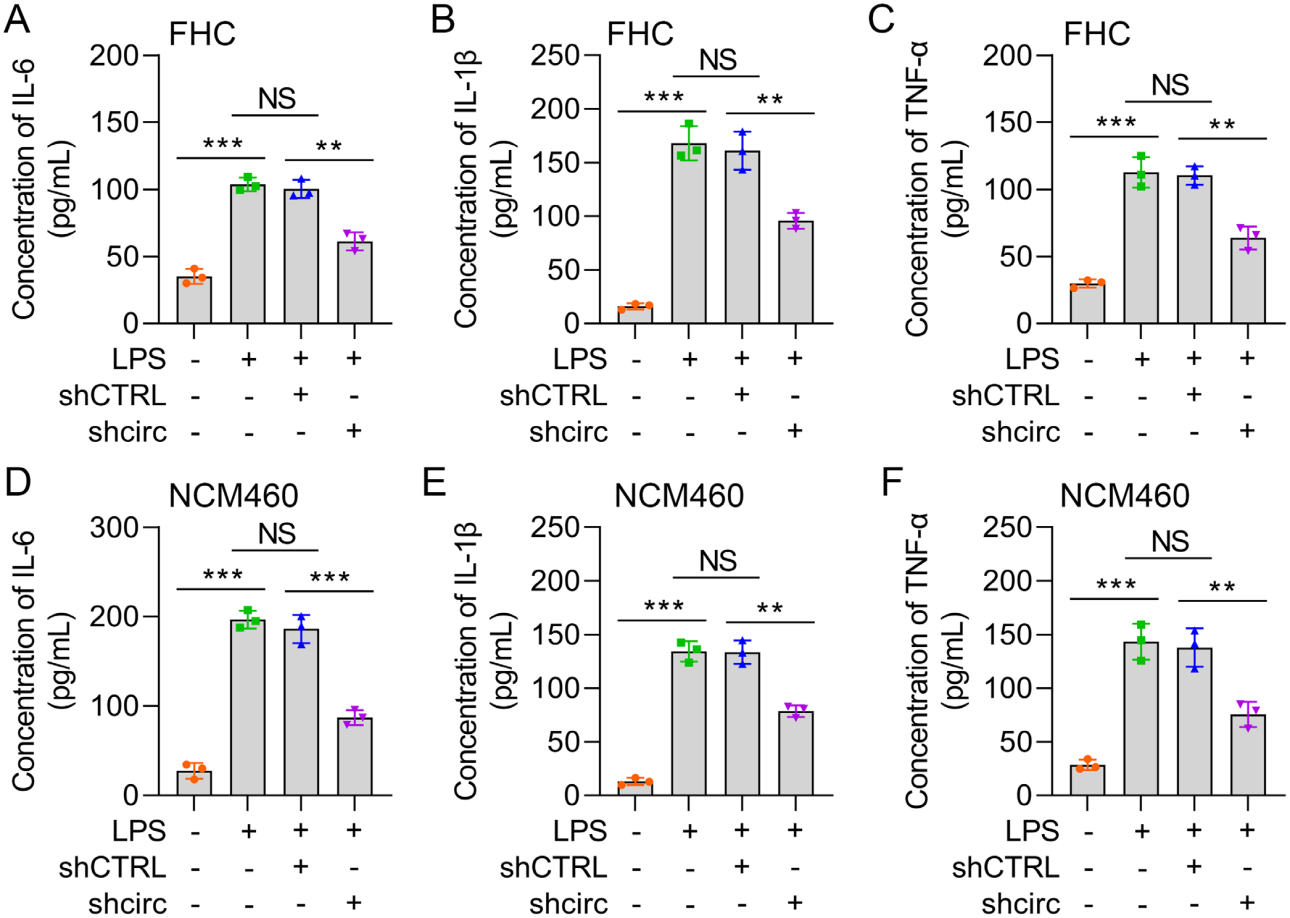
Hsa_circ_0004662 augments LPS‐stimulated inflammatory cytokine release. (A–C) IL‐6, IL‐1β and TNF‐α levels in LPS‐, LPS + shCTRL‐ and LPS + shcirc‐treated FHC cells were determined via ELISA. (D–F) IL‐6, IL‐1β and TNF‐α contents in LPS‐, LPS + shCTRL‐ and LPS + shcirc‐induced NCM460 cells were determined via ELISA. The data are presented as the means ± SDs (*n* = 5); **p < 0.01, ***p < 0.001.

Intestinal inflammation is directly related to the secretion of proinflammatory mediators. Therefore, this study investigated whether downregulating hsa_circ_0004662 in an LPS‐stimulated EC model reduces inflammation. Next, the sh‐circ_0004662 vector was incorporated into FHC and NCM460 cells to deplete circ_0004662 expression, followed by 24 h of LPS (10 μg/mL) induction. As shown in Figure 2A, circ_0004662 expression was substantially reduced in the LPS + sh‐circ_0004662 cohort, suggesting that the synthetic sh‐circ_0004662 vector was effective. Next, circ_0004662 downregulation promoted cell proliferation (Figure 2B–D). Furthermore, the FCM assay revealed that the downregulation of circ_0004662 suppressed the cell apoptosis caused by LPS (Figure 2E,F). Furthermore, the FHC and NCM460 cells transfected with sh‐circ0004662 presented lower levels of the apoptosis‐related proteins Cas3 and Cas9 than the sh‐CTRL cells did (Figure 2G and Figure S1). However, downregulation of hsa_circ_0004662 inhibited the release of the inflammatory factors IL‐6, IL‐1β and TNF‐α in the LPS‐stimulated EC model (Figure 3A–F). These results suggested that the downregulation of circ_0004662 increased cell proliferation and repressed cell apoptosis in LPS‐treated FHC and NCM460 cells. Importantly, downregulation of hsa_circ_0004662 could suppress LPS‐induced inflammatory cytokine secretion.

### 3.3 Hsa_circ_0004662 as a Sponge of miR‐532

**FIGURE 4:**
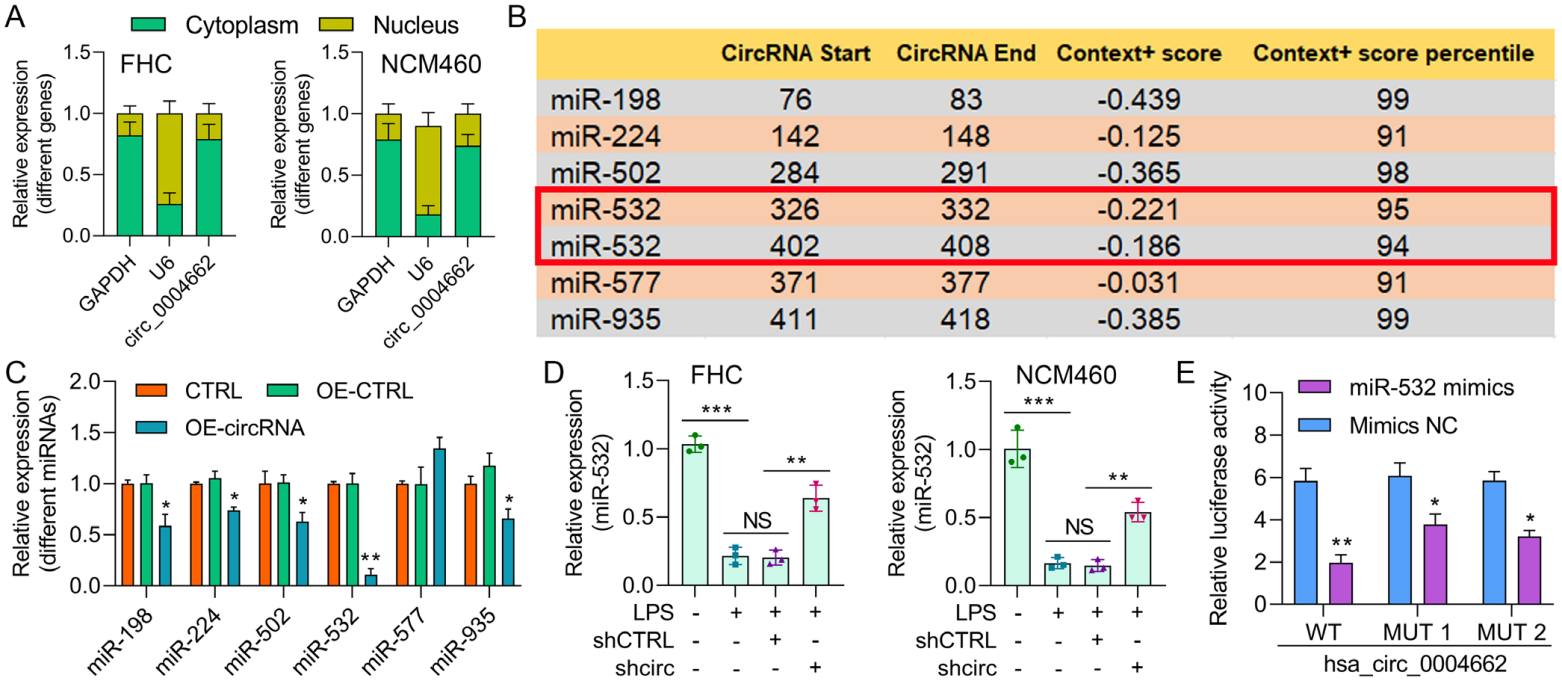
Direct interaction of circ_0004662 with miR‐532. (A) Quantification of the nuclear and cytoplasmic circ_0004662 fractions via RT‐qPCR analysis. (B) Identification of miRNAs related to hsa_circ_0004662 via the CircInteractome database. (C) The levels of different miRNAs were determined via qPCR after the overexpression of hsa_circ_00046622, and the strongest miRNA candidate was miR‐532. (D) Evaluation of miR‐532 expression in LPS‐, LPS + shCTRL‐ and LPS + shcirc‐treated FHC and NCM460 cells via RT‐qPCR. (E) Luciferase activity of FHC and NCM460 cells coexpressed with circ_00046622 WT or circ_00046622 MUT and with miR‐532 or miR‐NC, as detected via a dual‐luciferase reporter (DLR) assay. The data are presented as the means ± SDs of independent triplicate experiments; ***p < 0.001.

Segmentation analysis between the cytoplasm and nucleus confirmed that circ0004662 was found primarily in the cytoplasm rather than in the nucleus. Next, RT‐qPCR was used to evaluate the nuclear and cytoplasmic RNA fractions to determine the localization of circ_0004662 in FHC and NCM460 cells. The acquired data revealed that circ_0004662 was primarily located within the cytoplasm of FHC and NCM460 cells (Figure 4A), suggesting that circ_0004662 primarily affects the cytoplasm of EC cells. According to the CircInteractome database, miR‐198, miR‐224, miR‐502, miR‐532, miR‐577 and miR‐935 are the miRNAs that interact with hsa_circ_0004662 (context+ score percentile > 90; Figure 4B). After the overexpression of hsa_circ_00046622, the levels of several miRNAs were determined via qPCR. The results demonstrated that miR‐532 was the most promising target miRNA (Figure 4C). The effect of circ_0004662 on the expression of miR‐532 was subsequently investigated. As shown in Figure 4D, sh_circ_0004662 vector incorporation increased the miR‐532 level in FHC and NCM460 cells compared with that in the sh‐CTRL groups. Next, the DLR assay confirmed the targeting relationship between circ_0004662 and miR‐532. We demonstrated a pronounced reduction in circ_0004662 WT luciferase activity following miR‐532 transfection in FHC and NCM460 cells. However, the circ_0004662 MUT luciferase activity remained unchanged upon miR‐532 transfection (Figure 4E). These findings demonstrated that circ0004662 directly interacts with miR‐532 because it functions as a molecular sponge for miR‐532 in ECs.

### 3.4 HMGB3 is a Target Gene of miR‐532

**FIGURE 5:**
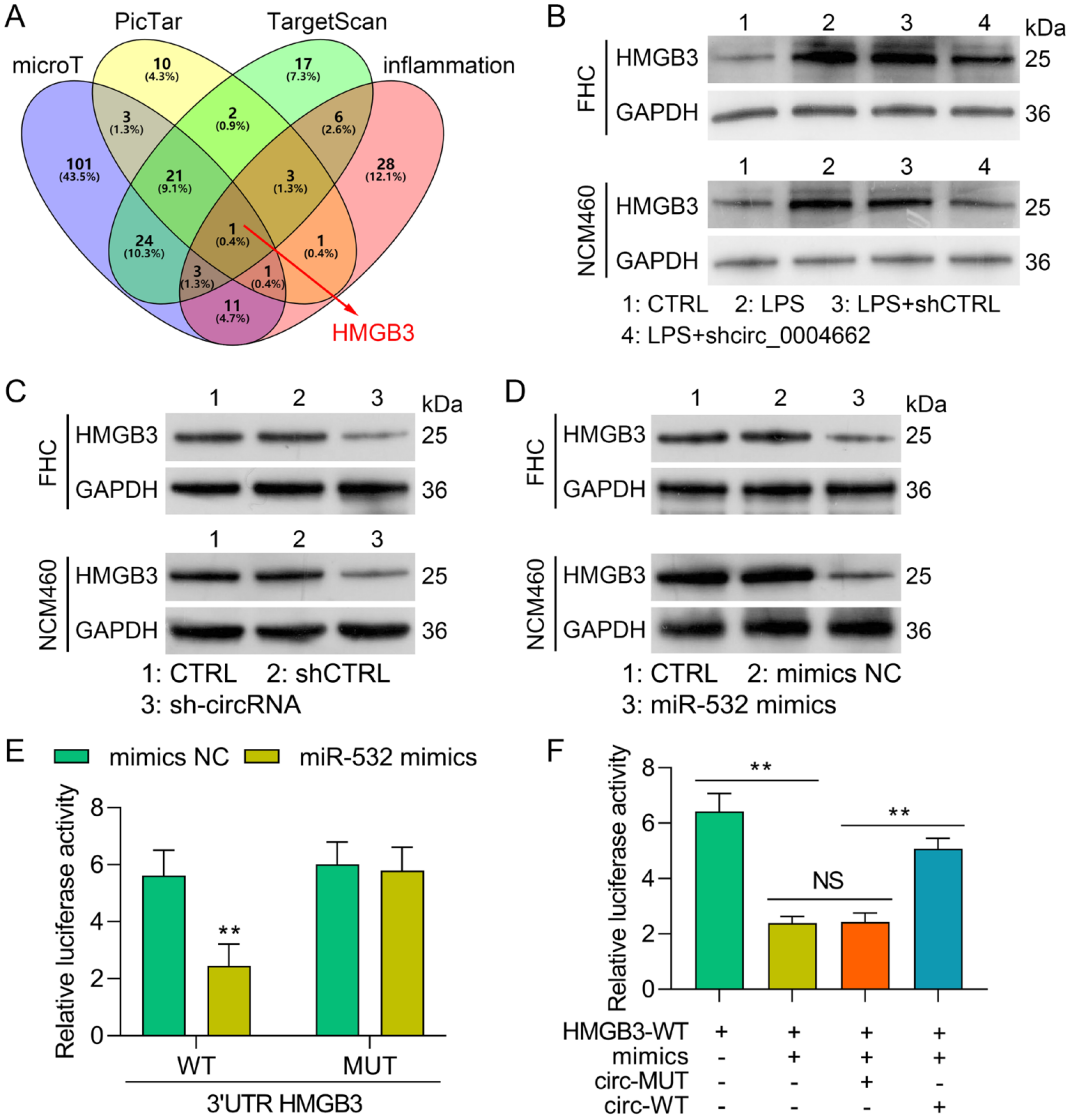
HMGB3 is a target of miR‐532. (A) In the PicTar, TargetScan and MicroT databases, the binding site between miR‐532 and HMGB3 is related to inflammation. (B) HMGB3 expression in LPS‐, LPS + shCTRL‐ and LPS + shcirc‐treated FHC and NCM460 cells via Western blot analysis. (C) Knockdown of circ_0004662 resulted in decreased HMGB3 levels according to Western blot analysis. (D) HMGB3 protein expression in FHC and NCM460 cells transfected with the miR‐532 control or miR‐532 mimic was also analysed via Western blot analysis. (E) Detection of interactions between miRNA‐532 and HMGB3A via a dual‐luciferase assay. (F) Relative luciferase activity in 293 T cells following coincorporation with the respective plasmids (with WT or mut circ_00046622), along with either the miR‐532 mimics or the control. The data are presented as the means ± SDs (*n* = 3); *p < 0.05.

The inflammation, PicTar, TargetScan and microT databases were used to identify miR‐532 downstream targets. A total of 232 potential targets were predicted to be regulated by miR‐532 (Figure 5A). However, the four databases predicted that only HMGB3 was a target of miR‐532. Furthermore, the incorporation of shRNA targeting circ_0004662 decreased the HMGB3 content in the LPS‐stimulated FHC and NCM460 cells compared with that in the LPS + shCTRL cells (Figure 5B). Next, we incorporated circ_0004662 into FHC and NCM460 cells and verified that circ_0004662 knockdown specifically reduced the amount of HMGB3 protein in FHC and NCM460 cells (Figure 5C). Moreover, the addition of the miR‐532 mimic drastically decreased the HMGB3 content in both FHC and NCM460 cells (Figure 5D), indicating that miR‐532 targets HMGB3. DLR assays suggested that the luciferase activity of the HMGB3 wild‐type vector may be considerably inhibited by miR‐532 overexpression but not that of the HMGB3 mutant reporter vector (Figure 5E). Elevated miR‐532 levels also significantly decreased HMGB3‐WT‐driven luciferase activity, which was restored by circ_WT but not circ_MUT (Figure 5F). Overall, HMGB3 was found to be a direct downstream effector of miR‐532.

### 3.5 circ_0004662 Suppresses LPS‐Stimulated Apoptosis and Inflammation by Modulating miRNA‐532/HMGB3

**FIGURE 6:**
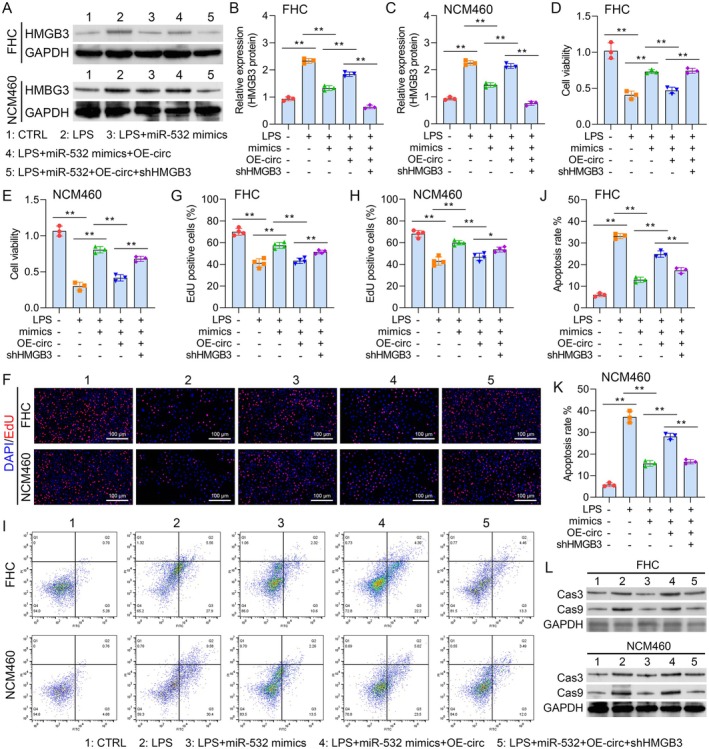
Circ_0004662 suppresses LPS‐stimulated apoptosis by modulating miRNA‐532/HMGB3. FHC and NCM460 cells were treated with LPS, LPS+ miR‐532 mimics, LPS+ miR‐532 mimics+OE‐circ or LPS+ miR‐532 mimics+ OE‐circ+shHMGB3. (A–C) WB analysis was used to verify the expression level of HMGB3. (D, E) A CCK‐8 assay was used to evaluate whether circ_0004662 regulated cell proliferation by modulating the miRNA‐532/HMGB3 axis. (F–H) An EdU assay was used to determine whether HMGB3 downregulation can alleviate the inhibitory effect of circ_0004662 on LPS‐stimulated models of EC proliferation via the targeting of miR‐532. (I–K) Detection of the role of circ_0004662/miR‐532/HMGB3 in apoptosis in the respective models. (L) Investigation of the inflammatory effect in an LPS‐stimulated EC model via WB analysis. The data are presented as independent triplicate experiments and are presented as the means ± SDs from five replicates of individual samples: *p < 0.05, **p < 0.01.

**FIGURE 7:**
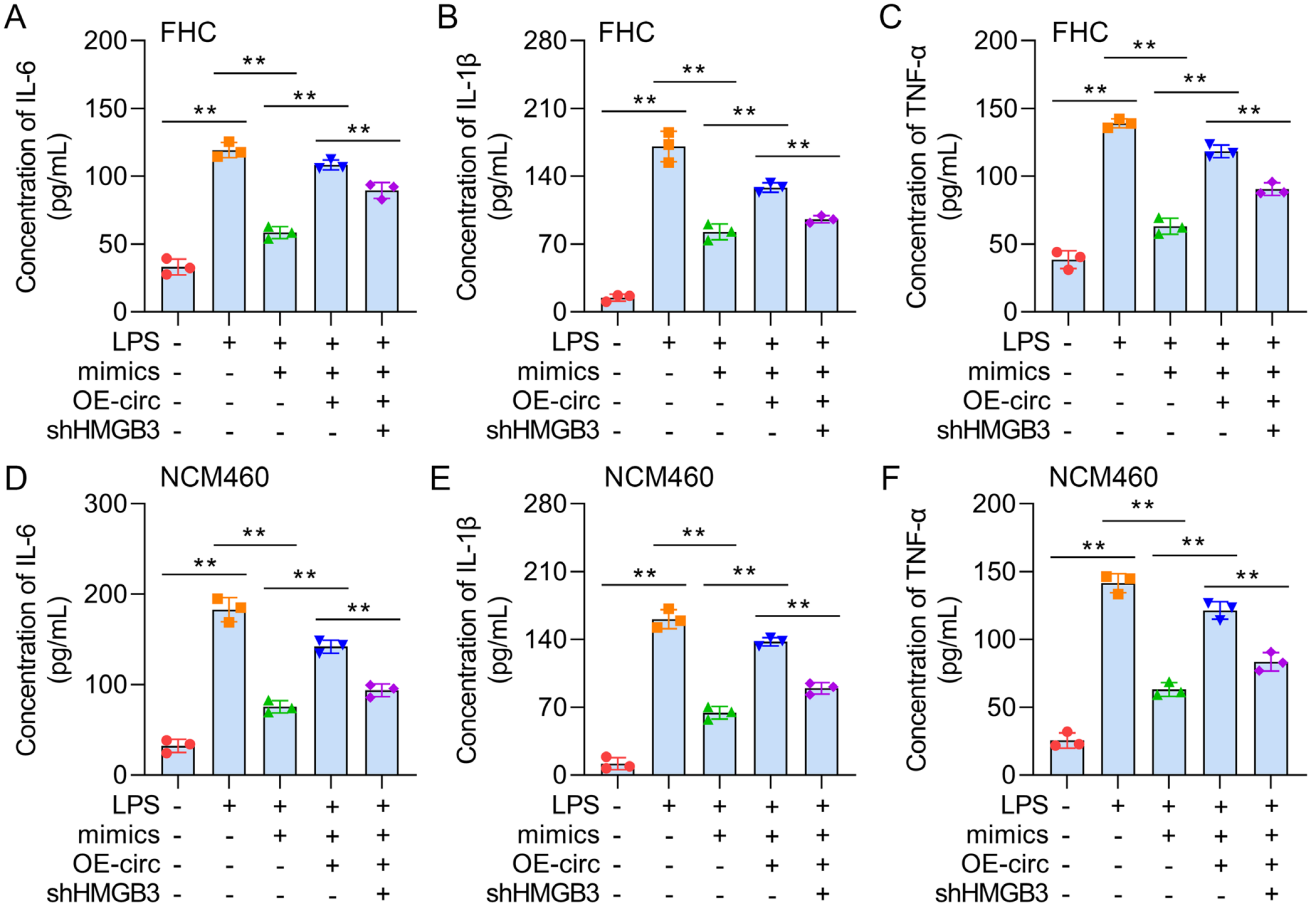
Circ_0004662 suppresses LPS‐stimulated inflammation by modulating miRNA‐532/HMGB3.

To explore the functions of the circ_0004662‐miR‐532‐HMGB3 axis, FHC and NCM460 cells were exposed to LPS, LPS + miR‐532 mimics, LPS + miR‐532 mimics+OE‐circ or LPS+ miR‐532 mimics+OE‐circ+shHMGB3. Western blot analysis revealed that the overexpression of miR‐532 significantly reduced the expression of HMGB3. However, circ_0004662 reversed the reduction in the protein level of HMGB3 caused by miR‐532 overexpression (Figure 6A–C). Both CCK‐8 and EdU analyses demonstrated that ectopic miR‐532 expression increased LPS‐stimulated EC proliferation. However, the upregulation of circ_0004662 inhibited the proliferation ability caused by miR‐532 overexpression, whilst HMGB3 depletion abrogated the suppressive effect of circ_0004662 on LPS‐stimulated EC model proliferation (Figure 6D–H). Consistent with these findings, FCM analysis revealed that ectopic miR‐532 expression suppressed LPS‐stimulated EC apoptosis. However, upregulation of circ_0004662 facilitates the apoptosis process caused by miR‐532 overexpression, and HMGB3 downregulation inhibits the effect of circ_0004662 upregulation on LPS‐induced FHC apoptosis (Figure 6I–K). The Western blot results revealed that miR‐532 overexpression decreased the levels of Cas‐3 and Cas‐9; however, upregulation of circ_0004662 rescued Cas‐3 and Cas‐9 expression caused by miR‐532 overexpression. In addition, HMGB3 suppression decreased the levels of Cas‐3 and Cas‐9, which reversed the accelerative effect of hsa_circ_0004662 upregulation on the expression of these proteins (Figure 6L and Figure S2). Moreover, the ELISA results revealed that ectopic miR‐532 expression suppressed LPS‐stimulated IL‐1β, IL‐6 and TNF‐α secretion, whereas upregulation of circ_0004662 reversed this phenomenon. However, HMGB3 downregulation suppressed the ability of hsa_circ_0004662 to promote IL‐1β, IL‐6 and TNF‐α secretion in LPS‐induced FHC and NCM460 cells by regulating miR‐532 expression (Figure 7). These findings demonstrated that circ_0004662 suppresses LPS‐stimulated apoptosis and inflammation by modulating miRNA‐532/HMGB3.

### 3.6 Suppression of circ_0004662 Improves DSS‐Stimulated UC in Mice

**FIGURE 8:**
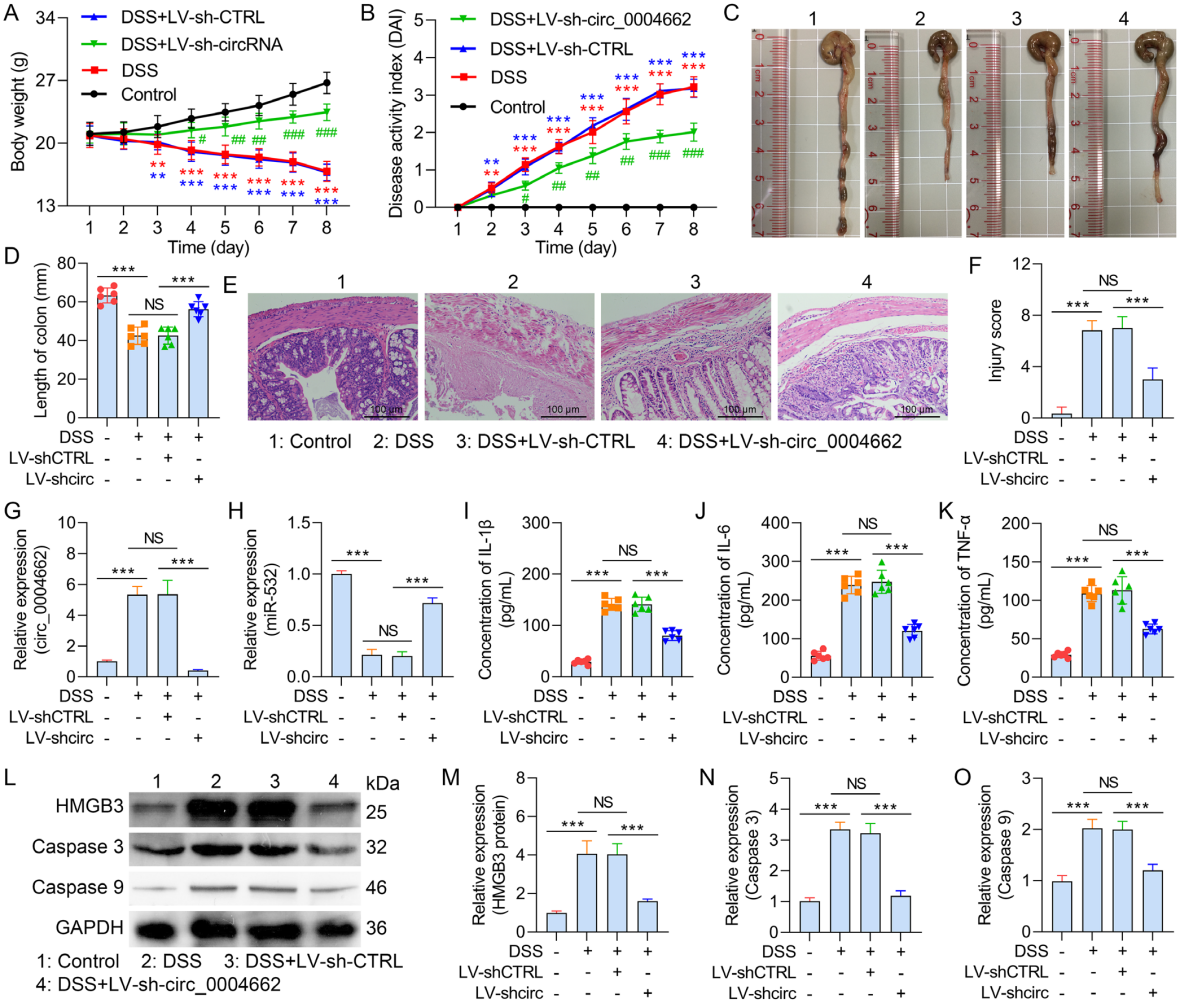
Hsa_circ_0004662 downregulation attenuated DSS‐induced acute ulcerative colitis in mice. (A) Body weight loss after DSS administration was significantly repressed after sh‐circ_0004662 treatment. (B) Disease activity index (DAI) scores for the experimental procedures in mice. (C, D) sh‐circ_0004662 treatment reduced the loss of colon length caused by DSS treatment. (E) Representative images of HE staining; scale bar = 100 μm. The control mice presented normal mucosa; the DSS and DSS + LV‐sh‐CTRL groups presented mucosa atrophy and villus loss with degeneration and necrosis of epithelial cells and infiltration of inflammatory cells and the DSS + LV‐sh‐circ_0004662 group presented better morphological structure and infiltration of fewer inflammatory cells. (F) Injury scores of the five groups. (G, H) circ_0004662 and miR‐532 expression was measured via qRT‐PCR analysis. (I–K) Circ_0004662‐mediated regulation of IL‐1β, IL‐6 and TNF‐α secretion in DSS‐exposed mice via ELISA analysis. (L) WB images. (M–O) Detection of HMGB3, caspase 3 and 9 levels via WB. The data are presented as the means ± SDs for each sample (*n* = 5); **p < 0.01, ***p < 0.001, ^#^ *p* < 0.05, ^##^ *p* < 0.01, ^###^ *p* < 0.001.

The mice received 5 days of 3% DSS for UC induction. Compared with the control cohort, the DSS + LV‐sh‐CTRL‐ and DSS‐alone‐treated cohorts experienced much smaller weight alterations. After 3 days, the weight substantially increased. On Day 8, there was no change in the weight of the mice in the DSS + LV‐sh‐CTRL group. Furthermore, the weight of the DSS + LV‐sh‐circRNA group mice was much lower than that of the DSS‐alone and DSS + LV‐sh‐CTRL groups (Figure 8A). Both the DSS‐alone and DSS + LV‐sh‐CTRL cohorts presented considerably higher DAI scores than the control cohort (Figure 8B). The DSS + LV‐sh‐circRNA cohort presented a lower DAI score than the DSS‐alone and DSS + LV‐sh‐CTRL cohorts did. Compared with control mice, DSS‐treated mice presented considerably shorter colons. Moreover, circ_0004662 downregulation abrogated DSS‐stimulated colon length shortening (Figure 8C,D). HE staining was used to evaluate colonic injury. The results demonstrated that the DSS + LV‐sh‐circRNA group had significantly reduced DSS‐induced submucosal edema, inflammatory cell infiltration and loss of crypts and ECs (Figure 8E,F). Furthermore, the qPCR results confirmed that circ_0004662 expression was markedly reduced in the DSS + LV‐sh‐circRNA cohort (Figure 8G). Moreover, qPCR studies revealed that in DSS‐induced UC model mice, transfection with sh_circ0004662 substantially increased miR‐532 expression (Figure 8H). DSS treatment subsequently increased IL‐1β, IL‐6 and TNF‐α production. Conversely, compared with that in the DSS‐alone and DSS + LV‐sh‐CTRL groups, inflammatory cytokine production was considerably lower in the DSS + LV‐sh_circ_0004662 group (Figure 8I–K). The Western blot results revealed that circ_0004662 suppression decreased the levels of HMGB3 and the major apoptosis‐associated colonic proteins, caspase‐3 and caspase‐9, in DSS‐induced UC mice (Figure 8L–O). Overall, the findings revealed that hsa_circ_0004662 downregulation prevented DSS‐stimulated morphological changes in colon tissue whilst preserving colon tissue.

## 4 Discussion

Ulcerative colitis (UC) is a persistent, nonspecific IBD characterised by inflammation and ulcerative alterations in the intestinal mucosa. Its lesions predominantly occur within the mucosal layer, the submucosal layer of the colon and the rectal region [4, 15]. However, the main mechanism of its development has not yet been fully explored. Studies indicate that a dysfunctional immune system has a significant role in UC‐associated intestinal inflammation and tissue damage. In contrast, other studies have indicated that they may be associated with genetic, environmental, infectious and immune factors. Its clinical treatment typically involves the administration of immunosuppressants, corticosteroids and aminosalicylates [16, 17, 18]. In this study, circ_0004662 was extensively expressed in UC patients and colitis animal models. Furthermore, the expression of hsa_circ_0004662 and HMGB3 was negatively associated with LPS‐induced ECs. The expression of HMGB3 and the recruitment of the inflammatory factors IL‐6, IL‐1β and TNF‐α were reduced by hsa_circ_0004662 knockdown. Therefore, hsa_circ_0004662 induces the apoptosis of colonic ECs stimulated with LPS by targeting HMGB3. Hsa_circ_0004662 can also promote murine DSS‐induced colitis, indicating its efficacy in UC therapy.

Growing evidence indicates that circRNAs are crucial in UC. In fact, they have been identified as potential indicators. The involvement of circRNA‐SOD2 in UC has been documented [19]. Xu et al. [11] demonstrated that circRNA‐HECTD1 enhances autophagy and influences UC. Conversely, circRNA‐102,610 is expressed at increased levels in UC and allows epithelial–mesenchymal transition via *the action of* miR‐130a‐3p [20]. However, few studies have explored the activity of hsa_circ_0004662 in UC. This study revealed that hsa_circ_0004662 was altered in UC. These results enhance our current knowledge of circRNAs and their action in UC, potentially aiding in elucidating the physiological processes underlying UC.

Multiple reports have suggested that circRNAs modulate disease mechanisms through miRNAs [21, 22, 23]. Many miRNAs play a significant role in UC [24, 25]. Li [26] reported that miR‐146a and miR‐196 are intricately linked to UC. Wu et al. [27] demonstrated that miR‐223‐3p reduces UC in *a* pyroptosis‐dependent fashion. Conversely, miR‐21 and miR‐155 inhibit UC [28]. Previous investigations revealed that the level of miR‐532‐3p is consistently increased in CD patients compared with healthy individuals [29, 30]. Recently, Dinesh et al. [31] reported that miR‐532‐3p functions as an antagonist in LPS/TNF‐α‐induced macrophages via its interaction with the ASK1/p38 MAPK axis, thereby inhibiting the inflammatory response caused by this axis. Therefore, it is a target candidate for the management of autoimmune inflammatory conditions. The current experimental data confirmed that miRNA‐532 is a downstream target of hsa_circ_0004662, with positively identified binding sites. The analysis revealed significant suppression of miRNA‐532, which was influenced by the level of hsa_circ_0004662 in UC. These findings also demonstrated that hsa_circ_0004662 silencing suppressed cell apoptosis and inflammatory reactions in LPS‐treated ECs by decreasing the level of HMGB3.

HMGB3 has previously been shown to play crucial roles in human diseases, including breast cancer (BC) [32], glioblastoma GBM [33], silica‐induced pulmonary inflammation [34], and myocardial infarction (MI) [35]. Zhou et al. reported that CircRNA_102179 regulates the miR‐330‐5p/HMGB3 network, thereby augmenting non‐small cell lung cancer cell proliferation, dissemination and penetration [36]. Similarly, Xiao et al. [37] revealed that Circ_CLIP2 knockdown inhibits glioma progression by interacting with miR‐195‐5p/HMGB3. This study also confirmed that HMGB3 serves as a direct target of miR‐532, which negatively controls its function in LPS‐stimulated ECs. Our findings indicated that the miR‐532 mimic relieved LPS‐induced EC injury by targeting HMGB3, highlighting the remarkable role of HMGB3 in the progression of UC.

We conducted the first study to evaluate hsa_circ_0004662 expression in UC and explore the molecular mechanism associated with its function, particularly in UC development and progression. Our findings pave the way for a more accurate theoretical foundation for UC pathogenesis and suggest possible targets for its management. However, some limitations remain. For instance, hsa_circ_0004662 may exert its effects on UC through the modulation of additional signalling pathways beyond the miR‐532/HMGB3 axis. Identifying and characterising these pathways is crucial for a more comprehensive understanding of its role in UC, and this will be a key focus of future investigations. Additionally, mitochondrial dysfunction is well‐established as a critical driver of apoptosis, particularly under LPS‐induced stress. Whilst our results demonstrate that hsa_circ_0004662 knockdown inhibits apoptosis by downregulating Caspase‐3 and Caspase‐9, we did not examine its potential effects on mitochondrial morphology and function. This represents another limitation of the current study. Future research should address whether hsa_circ_0004662 knockdown can restore mitochondrial integrity, thereby providing further mechanistic insights into its anti‐apoptotic effects. Investigating these aspects will enhance our understanding of the molecular pathways regulated by hsa_circ_0004662 and its therapeutic potential in UC management. Moreover, our findings revealed that Hsa_circ_0004662 knockdown enhances cell proliferation, as evidenced by the results of CCK‐8 and EdU assays. However, this study did not investigate whether Hsa_circ_0004662 directly influences cell cycle regulation. Given the close relationship between the cell cycle and cellular proliferation, future studies are warranted to explore how Hsa_circ_0004662 might modulate specific cell cycle phases or checkpoints. This limitation highlights an area for further research that could deepen our understanding of the mechanisms by which Hsa_circ_0004662 contributes to UC progression and provide a more comprehensive view of its biological role.

## 5 Conclusions

In summary, this investigation revealed that circ_0004662 suppresses the UC process by modulating miRNA‐532/HMGB3. These results indicate that circ_0004662/miRNA‐532/HMGB3 represents a new management strategy for UC patients.

## Author Contributions

**Chunhua Qiu:** data curation (equal), investigation (equal), writing – review and editing (lead). **Yun Chen:** data curation (lead), methodology (equal), visualization (equal), writing – review and editing (equal). **Huan Xia:** data curation (lead), investigation (equal), visualization (equal), writing – original draft (lead), writing – review and editing (equal). **Jun Duan:** formal analysis (equal), writing – review and editing (equal). **Lu Zhang:** investigation (equal), writing – review and editing (equal). **You Zhang:** methodology (equal), writing – review and editing (equal). **Ziyang Chen:** conceptualization (equal), funding acquisition (lead), writing – review and editing (equal). **Li Zhang:** conceptualization (equal), formal analysis (equal), funding acquisition (equal), project administration (equal), writing – review and editing (equal).

## Ethics Statement

Ethical approval was obtained from the appropriate institutional board, and the study adhered to the guidelines of the Declaration of Helsinki (2013 revision). Informed consent was obtained from individual participants prior to tissue sample collection. And the Animal Care and Use Committee of the participating institution provided approval for all the animal protocols [Lunshen(Yan)2022‐388].

## Conflicts of Interest

The authors declare no conflicts of interest.

## Supporting information


Data S1.

## Data Availability

The datasets generated during and/or analysed during the current study are available from the corresponding author on reasonable request.
