# Supplementary material for: Hsa_circ_0004662 Accelerates the Progression of Ulcerative Colitis via the microRNA‐532/HMGB3 Signalling Axis
Source: J Cell Mol Med. 2025 Mar 18;29(6):e70430. doi: 10.1111/jcmm.70430 (PMC11916553; doi:10.1111/jcmm.70430)
Supplement: Supplementary file 1 — Data S1. [file JCMM-29-e70430-s001.docx]

Supplementary Materials for

**Hsa_circ_0004662 accelerates the progression of ulcerative colitis *via* the microRNA-532/HMGB3 signaling axis**

Chunhua Qiu *et al.*

* Correspondence to: Li Zhang and Ziyang Chen, Email: [1262867096@qq.com](mailto:1262867096@qq.com) (Li Zhang) and [czy780331@163.com](mailto:czy780331@163.com) (Ziyang Chen).

**This file includes:**

Figure. S1 to Figure. S2

Supplemental materials and methods


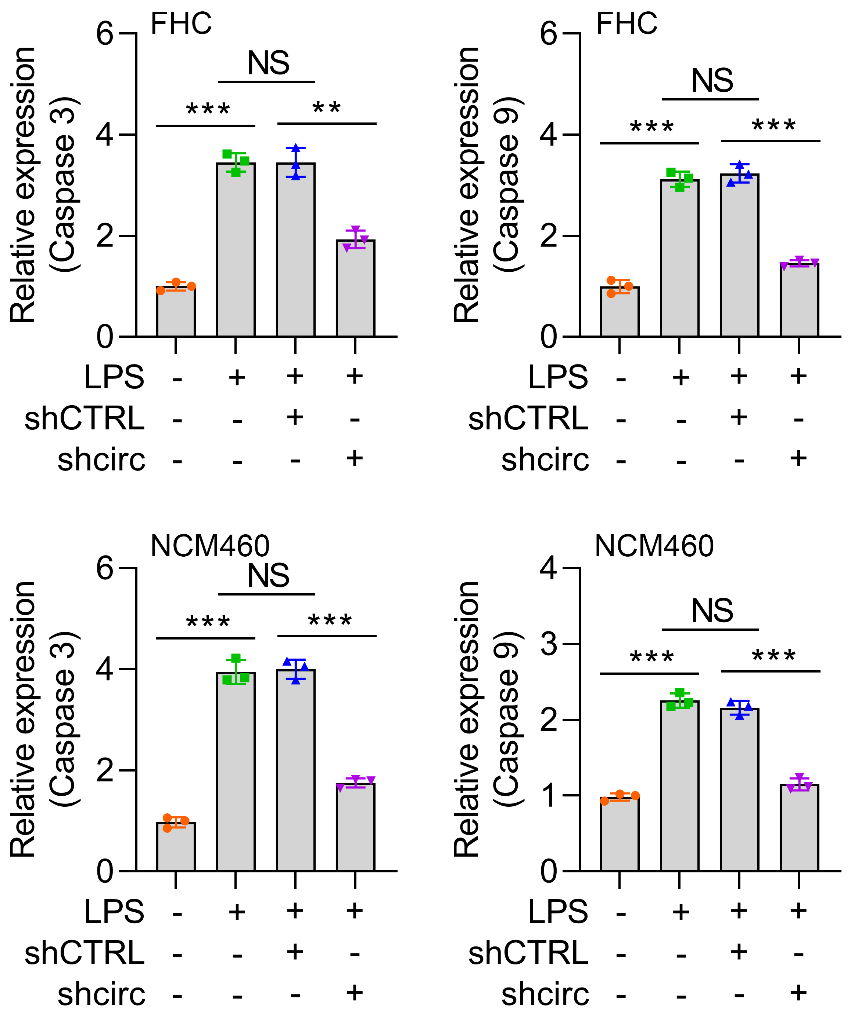


**Figure S1.** Caspase 3 and 9 protein levels in LPS-, LPS+shCTRL-, and LPS+sh-SLC6A14-treated FHC and NCM460 cells determined via western blot analysis. Gene expression was normalized to that of GAPDH (reference protein). ***p* < 0.01, ****p* < 0.01.


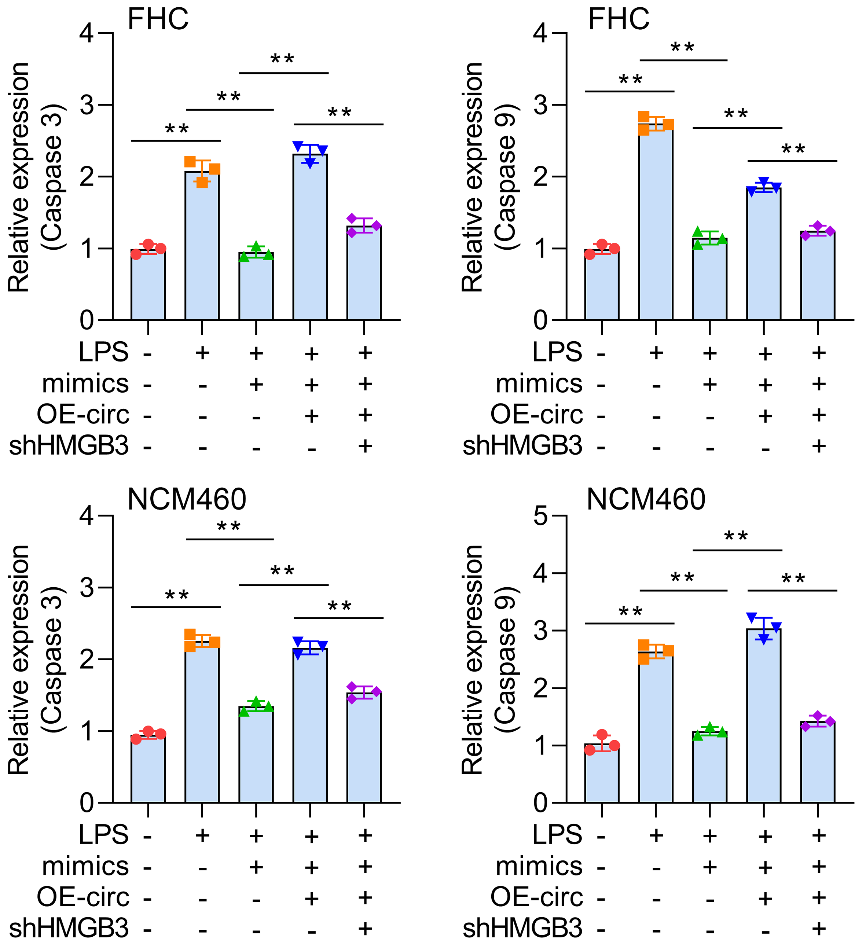


**Figure S2.** Western blot analysis of the expression of Caspase 3 and 9 in FHC and NCM460 cells exposed to LPS, LPS+miR-532 mimics+OE_circ_0004662 or LPS+miR-532 mimics+OE_circ_0004662+shHMGB3, followed by quantitative analysis. Gene expression was normalized to that of GAPDH (reference protein). ***p* < 0.01.

**Supplemental materials and methods**

**Cell culture**

The normal colon EC lines FHC and NCM460 were obtained from the National Collection of Authenticated Cell Cultures (Shanghai, China). RPMI-1640 (Gibco, USA) media supplemented with 10% fetal bovine serum (FBS; Gibco, USA) was used for FHC and NCM460 cell culture. As described in previous studies ^1,2^, the cells were exposed to LPS (10 ng/mL) for 6 h to establish a cell model of colitis. The cells were maintained in a 6-well plate in a humid chamber at 37 °C and 5% CO_2_.

**Plasmid synthesis and cellular incorporation**

Knockdown vectors, including short hairpin RNA targeting hsa_circ_0004662 (sh-hsa_circ_0004662) and a negative control (sh-negative control), as well as overexpression vectors comprising miR-532 agomir, agomir-negative control (agomir-NC), hsa_circ_0004662 (OE-hsa_circ_0004662) overexpression, and negative control (OE-NC) overexpression, in addition to low expression vectors (sh-HMGB3), were acquired from Synbio Technologies Co. Ltd. (Suzhou, China). Upon reaching approximately 70% confluence, the cells were plasmid-incorporated with Lipofectamine™ 2000, in line with the manufacturer’s recommendation (Thermo Scientific, Massachusetts, USA). Next, RNA isolation was performed at 48 h after incorporation, and protein isolation was performed at 72 h after incorporation.

**CCK-8 assessment**

Cell proliferation was examined with a Cell Counting Kit-8^®^ (CCK-8; Dojindo Laboratories™, Japan). Approximately 2 × 10^3^ cells were grown in a 96-well plate (triplicates) and incubated for 120 min at 37 °C. The absorbance (OD) was recorded at 450 nm at 24 h intervals for 72 h.

**EdU evaluation**

A 5-ethynyl-20-deoxyuridine (EDU) assay kit^®^ (Ribobio™, China) was used to assess cell proliferation. Cultures were grown in confocal plates (10 × 10^5^ cells/well), followed by incubation with 50 μM EDU buffer (37 °C/120 min), fixation with 4% formaldehyde (30 min), and permeabilization with 0.1% Triton X-100 (20 min). After the addition of EdU to the culture medium, the cell nuclei were subjected to Hoechst staining, followed by observation *via* fluorescence microscopy.

**Enzyme-linked immunosorbent assay (ELISA)**

FHC and NCM460 cells (5 × 10^5^/well) were trypsinized, centrifuged (300 × g for 5 min) at room temperature (RT) and collected. Following washing with PBS, the cells were lysed and spun at 300 × g for 10 min at RT. The inflammatory mediator contents, specifically those of TNF-α, IL-1β, and IL-6, were assessed in all groups of FHCs and NCM460 cells via Simple Step ELISA^®^ kits (Cat # ab181421, ab229384, and ab178013; Abcam, Cambridge, UK), as per the guidelines provided by the manufacturer. The absorbance (OD) was assessed at 450 nm *via* a microplate reader.

**Gene expression *via* qRT‒PCR**

The tissues and cells were harvested, and the total RNA content was extracted with TRIzol (Invitrogen, USA). cDNA synthesis was then performed via the Promega GoScript RT system (Promega Madison, WI, USA). A 20 µL cDNA mixture was subsequently used for PCR amplification on an ABI7500 real-time quantitative PCR apparatus. First-strand cDNA for miRNAs was synthesized *via* a cDNA synthesis kit (R601, Novabio, Shanghai, China) and the stem‒loop method ^3^. Alterations in the relevant relative gene profiles were determined *via* the 2^−ΔΔCt^ formula.

**Western blotting**

After tissue harvesting, total proteins were isolated with RIPA buffer (Beyotime, Guangzhou, China) and a protease inhibitor cocktail (Roche, IN, USA). The cell lysate was loaded into 10% SDA‒PAGE for protein separation prior to transfer to PVDF membranes (Millipore, MA, USA), which were then blocked for 1 h in a blocking solution (5% nonfat milk in TBST) at room temperature. Next, the blot was incubated with specific antibodies against cleaved caspase 3 (poteintech, Cat No: 68773-1-Ig, 1:20000), caspase 9 (boster, BM4619, 1:1000), HMGB3 (poteintech, Cat No: 27465-1-AP, 1:1000), and GAPDH (poteintech, Cat No: 60004-1-Ig, 1:200000) for 24 h at 4 °C. Goat anti-rabbit IgG H&L (HRP) (Proteintech, Cat No: SA00001-2, 1:3000) or goat anti-mouse IgG H&L (HRP) (Proteintech, Cat No: SA00001-1, 1:3000) was used for 1-h blot probing at room temperature. Following washing and immersion in developer solution, protein bands were visualized on X-ray film.

**Subcellular fractionation**

Using the PARIS Kit (Invitrogen), we isolated the nuclear and cytoplasmic fractions from FHC and NCM460 cells in accordance with the provided instructions. The abundance of Circ_0004662 in both fractions was evaluated via qPCR, and *U6* and *GAPDH* served as the controls for the nuclear and cytoplasmic transcripts, respectively.

**Dual-luciferase reporter (DLR) gene assay**

We identified candidate miRNA docking sites in circ_0004662 via a web-based tool (<https://circinteractome.nia.nih.gov/>). Next, we developed luciferase reporter constructs for both circ_0004662 and relevant miRNAs. HEK293T cells were transformed with WT or mutant circ_0004662 reporter plasmids prior to transfection with the corresponding miRNA mimic or control constructs. Firefly and Renilla luciferase activities were evaluated at 48 h postincorporation, and the results are expressed as the firefly to Renilla luciferase activity ratio.

DIANA-microT (<http://diana.cslab.ece.ntua.gr/microT/>), PicTar (<http://www.pictar.org/>) and TargetScan (<http://www.targetscan.org>) were used to analyze the potential targets of miR-532. Next, we developed luciferase reporter constructs for both the 3’UTR of HMGB3 and relevant miRNAs. HEK293T cells were transformed with WT or mutant HMGB3 reporter plasmids prior to transfection with the corresponding miRNA mimic or control constructs. Firefly and Renilla luciferase activities were evaluated at 48 h postincorporation, and the results are expressed as the ratio of Renilla luciferase activity to firefly luciferase activity.

**Flow cytometry**

Apoptotic rates were evaluated *via* flow cytometry. The transfected cells were counted 48 h after incubation, grown in 96-well plates, and trypsinized with trypsin without EDTA. The cells were then subjected to washing with chilled PBS and centrifuged, and the supernatant was discarded. After that, the cellular apoptotic rate was measured with an annexin V–fluorescein isothiocyanate (FITC) apoptosis discovery kit (BioLegend, Inc.). The cells were subjected to resuspension in buffer (1X binding buffer, 100 μL). A 15-min treatment of the resuspended cells was carried out with 5 μL of propidium iodide (PI) and annexin V–FITC at RT (in the dark). Finally, the number of double-stained cells was assessed *via* a FACSCalibur FC instrument (BD Biosciences, San Jose, CA, USA). Apoptotic cell quantification was performed *via* v2.9 of CellQuest (BD Biosciences).

**Evaluation of DAI scores**

The UC degree of the mice was determined every day on the basis of gross rectal bleeding, body weight, and stool consistency. The severity of the disease was quantified by calculating a DAI score according to a published methodology. DAI = (weight loss score + stool characteristic score + occult blood score)/3.

**Colon histopathology**

Colon length was monitored overall, and 0.5 cm tissue sections were subjected to 24-h fixation in formalin (10%) before they were paraffin-embedded, sectioned (5 μm), and hematoxylin-eosin (H&E)-stained for histological examination. Individual sections were examined at 100x.

**Reference**

1. Liu C, Yan X, Zhang Y, et al. Oral administration of turmeric-derived exosome-like nanovesicles with anti-inflammatory and pro-resolving bioactions for murine colitis therapy. *Journal of nanobiotechnology*. Apr 29 2022;20(1):206. <https://doi.org10.1186/s12951-022-01421-w>.

2. Gu Q, Xia H, Song YQ, et al. SLC6A14 promotes ulcerative colitis progression by facilitating NLRP3 inflammasome-mediated pyroptosis. *World journal of gastroenterology*. Jan 21 2024;30(3):252-267. <https://doi.org10.3748/wjg.v30.i3.252>.

3. Chen C, Ridzon DA, Broomer AJ, et al. Real-time quantification of microRNAs by stem-loop RT-PCR. *Nucleic Acids Res*. Nov 27 2005;33(20):e179. <https://doi.org10.1093/nar/gni178>.
